# Angiopoietins as biomarkers of schistosomiasis severity: a cross-sectional study

**DOI:** 10.1017/S0031182025000289

**Published:** 2025-03

**Authors:** Rachael Lima Sobreira Coimbra, Gdayllon Cavalcante Meneses, Rosângela Lima de Freitas Galvão, Marta Cristhiany Cunha Pinheiro, Luciana Maria de Oliveira, Nicole Coelho Lope, Bruna Viana Barroso Martins, Letícia Machado de Araújo, Rebeca Yasmin Ribeiro Vieira, Reinaldo Barreto Oriá, Alice Maria Costa Martins, Elizabeth de Francesco Daher, Ângela Maria da Silva, Fernando Schemelzer de Moraes Bezerra

**Affiliations:** 1 Faculdade de Medicina, Programa de Pós-Graduação em Ciências Médicas, Universidade Federal do Ceará, Fortaleza, CE, Brasil; 2 Departamento de Análises Clínicas e Toxicológicas, Laboratório de Bioprospecção Farmacêutica e Bioquímica Clínica, Universidade Federal do Ceará, Fortaleza, CE, Brasil; 3 Faculdade de Medicina, Programa de Pós-Graduação em Patologia, Universidade Federal do Ceará, Fortaleza, CE, Brasil; 4 Departamento de Análises Clínicas e Toxicológicas, Laboratório de Pesquisa em Parasitologia e Biologia de Moluscos, Universidade Federal do Ceará, Fortaleza, CE, Brasil; 5 Programa de Pós-Graduação em Ciências da Saúde, Universidade Federal de Sergipe, Sao Cristovao, SE, Brasil

**Keywords:** angiopoietins, biomarkers, parasitic load, schistosomiasis

## Abstract

Schistosomiasis, a parasitic disease caused by *Schistosoma* species, remains highly prevalent in tropical regions, where it contributes significantly to hepatic and vascular complications. Despite the well-established role of parasitic eggs in driving inflammation and organ damage, the specific vascular mechanisms remain poorly understood. Given the role of angiogenesis and vascular remodelling in tissue repair, the angiopoietins (ANGs) could be promising biomarkers to evaluate disease progression. This study aims to explore the relationship between ANG levels with parasitic load in patients with schistosomiasis. In this cross-sectional study, 126 schistosomiasis patients were stratified into three groups based on parasitic egg burden: negative, low, and moderate/high. Demographic, clinical, and laboratory data were collected, and serum ANGs were quantified via enzyme-linked immunosorbent assays. Parasitic load was assessed through stool examination, quantifying the number of *Schistosoma* eggs per gram of faeces. Additional clinical parameters, including liver abnormalities and blood chemistry, were evaluated. The ANG-2 levels and the ANG-2/ANG-1 ratio were significantly elevated in patients with higher egg burdens, particularly in the moderate/high group. The ANG-2/ANG-1 ratio was notably higher in patients with hepatosplenic schistosomiasis. While systemic blood pressure and oxygen saturation showed no significant differences between groups, patients with elevated triglycerides had lower ANG-2 levels. Elevated ANG-2 levels and an increased ANG-2/ANG-1 ratio correlate with higher parasitic burdens, reinforcing their potential as biomarkers for disease severity. These findings underscore the role of egg-induced inflammation in schistosomiasis pathophysiology and suggest that ANGs could aid in early diagnosis and treatment decisions, particularly in populations with high parasitic loads.

## Graphical abstract

**Figure:**
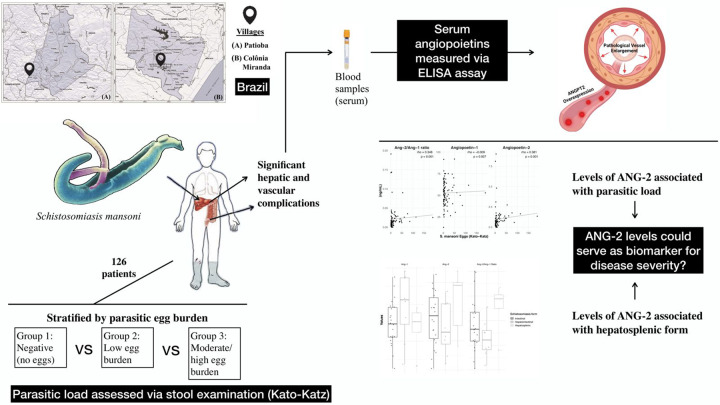

## Introduction

Schistosomiasis is one of the most prevalent neglected diseases in tropical and subtropical regions, affecting millions of people annually (Tamarozzi et al., 2021). *Schistosomiasis mansoni* infection is mostly found in 54 countries in Africa, Eastern Mediterranean and South America, especially in the Caribbean, Venezuela, and Brazil. In Brazil, 1·5 million people are estimated to live in areas at risk. From 2009 to 2019, the average positivity rate for *S. mansoni* in endemic areas of Brazil was 4·29%, with 423 117 cases detected from nearly 10 million tests (Brasil. Ministério da Saúde, 2024).

In schistosomiasis, the endothelial involvement in hepatosplenic cases, a severe form characterized by the enlargement of the liver and spleen, is significant and are directly impacted by the presence of *Schistosoma* eggs (Da Silva and Carrilho, 1992). These eggs, trapped in the hepatic vasculature causes inflammatory response and endothelial damage, leading to granuloma formation and fibrosis, resulting in increased vascular resistance and portal hypertension (Gryseels et al., 2006). Moreover, the release of pro-inflammatory cytokines and angiogenic factors, such as angiopoietins (ANGs) by the endothelial cells may perpetuates these processes.

ANGs, a family of vascular growth factors, have been studied as potential biomarkers for various inflammatory and vascular conditions (Lymperopoulou et al., 2015; Nicolini et al., 2019). ANG-1 and ANG-2 are crucial growth factors in the Tie system, which is essential for the development and maintenance of blood vessels. ANG-1 primarily acts as an agonist of the Tie2 receptor, promoting vascular stability (Gillen et al., 2019). It helps maintain the integrity of blood vessels, stimulates endothelial cell survival, reduces vascular permeability and also in the repair of blood vessels, playing a role in suppressing inflammation and regulating the endothelial barrier. In contrast, ANG-2 generally acts as a partial or competitive antagonist of ANG-1 at the Tie2 receptor and may destabilize vessels, increasing vascular permeability and facilitating inflammatory responses (Gillen et al., 2019). In inflammatory diseases, high ANG-2 levels reflect worsened vascular inflammation and increased mortality risk (David et al., 2012). Additionally, ANG-2 has been linked to chronic kidney disease and acute kidney injury progression (Araújo et al., 2019), an important clinical complication of Schistosomiasis.

However, there are no studies with ANGs in schistosomiasis patients. Thus, this present study aims to explore the role of ANGs, specifically ANG-2 and the ANG-2/ANG-1 ratio, in schistosomiasis severity, regardless of hepatic alterations. In this context, the role of ANGs could be a valuable for understand inflammatory and vascular processes which are relevant to the pathophysiology of schistosomiasis.

## Materials and methods

### Study design and population

We conducted a cross-sectional study from August 2022 to April 2023, involving patients diagnosed with schistosomiasis in two communities in the state of Sergipe, Brazil: The Patioba community in Japaratuba and the Colônia Miranda community in São Cristóvão. Patients were stratified into three groups based on their parasitic load: negative, low and moderate/high.

### Diagnosis of schistosomiasis and clinical data

The parasitological diagnosis of schistosomiasis was performed using the Kato–Katz method (Katz et al., 1972). Two stool samples were collected (on alternate days), and two slides were prepared from each. The intensity of infection by *S. manson*i was stratified in terms of eggs per gram of faeces (EPG) (WHO, 2002). In the study the patients were categorized into three groups: negative (patients with no eggs in the Kato–Katz method), low burden (EPG < 100) and moderate/high burden (EPG ≥ 100).

Data collection included sociodemographic characteristics such as age, sex, and race, as well as clinical parameters including systolic and diastolic blood pressure, oxygen saturation, and various hepatic and abdominal classifications. The changes and categorization of liver involvement were carried out according to the Niamey-Belo Horizonte protocol (Richter et al., 2000). Abdominal ultrasound was performed by a trained professional using a portable device C10RL from Konted®.

### Laboratory assessments and ANGs

Serum cholesterol (total and HDL) (expressed in mg/dL), triglycerides (TG) (expressed in mg/dL), direct bilirubin (BD) (expressed in ng/dL), total glutamic oxaloacetic transaminase (SGOT; TGO) and glutamic pyruvic transaminase (SGPT; TGP) (expressed in U/L) were measured using commercial kits from Bioclin® (Belo Horizonte, Brazil) and immunoturbidimetry in the BM-200 biochemical analyzer (Vyttra®, São Paulo, Brazil).

ANGs, ANG-1 and ANG-2 were measured using enzyme-linked immunosorbent assays. The assays was based on manufactured kits procedure (ANG-1: DY923, R&D systems^®^), and (ANG-2: DY623, R&D systems^®^). The ANG-2/ANG-1 ratio was calculated to evaluate balance of tie system mechanisms (Saharinen et al., 2008) possible correlations with parasitic load severity and clinical findings.

### Statistical analysis

Qualitative variables were expressed as absolute counts and percentages, and comparisons were made using the chi-square test. All quantitative variables were tested for normal distribution using the Shapiro–Wilk test. Normally distributed data were expressed as mean ± standard deviation, while non-normally distributed data were expressed as median and interquartile range. For comparisons between two independent groups, the Student’s t-test or Mann-Whitney test was used, depending on data normality. The parametric ANOVA test with Tukey’s post-hoc test was used for comparisons between three or more groups for normally distributed data, while the Kruskal–Wallis test with Dunn’s post-hoc test was used for non-normally distributed data. To determine correlations between the variables analysed, it was used Spearman’s Rho coefficient. The *P* < 0·05 was considered significant. All analysis were performed in SPSS for Macintosh (Version 23.0. Armonk, NY: IBM Corp).

## Results

In total, were included 126 schistosomiasis patients. Demographic, clinical, and laboratory parameters were evaluated among schistosomiasis patients, stratified by their parasitic load into negative, low, and moderate/high categories. From the initial cohort, patients were categorized into three groups: 30 patients with a negative parasitic load, 71 with a low load, and 25 with a moderate/high load. Notably, there was no significant age difference between the groups, with mean ages being 42·3 ± 12·7, 34·4 ± 15·5, and 33·2 ± 14·7 years, respectively. Racial composition significantly differed (*P* = 0·001), with a higher prevalence of mixed-race (Pardo) and Black (Preto) individuals in the moderate/high load group (Table 1).

**Table 1.** Sociodemographic and clinical characteristics related to the parasite load of patients with schistosomiasis

	Parasitic load			
	Negative (*n* = 30)	Low (*n* = 71)	Moderate/high (*n* = 25)	*p-value*
**Age (years)**	42·3 ± 12·7	34·4 ± 15·5	33·2 ± 14·7	0·054
**Sex**				0·578
Female	15 (50)	37 (52·1)	10 (40)	
Male	15 (50)	34 (47·9)	15 (60)	
**Skin color**				**0·001**
White	0 (0)	0 (0)	1 (4)	
Brown	0 (0)	10 (14·1)	15 (60)	
Black	0 (0)	6 (8·5)	3 (12)	
**Number of eggs (Kato–Katz)**	0 (0; 0)	4 (2; 6)	39 (20; 50)	**<0·001**
**Heart rate**	. (.; .)	81 (77; 83)	85 (70; 93)	0·197
**Systolic blood pressure**	. (.; .)	135 (121; 145)	137 (122; 150)	0·342
**Diastolic blood pressure**	. (.; .)	80 (73; 90)	80 (80; 90)	0·521
**Oxygen saturation**	. (.; .)	97 (96; 99)	98 (97; 99)	0·06
**Classification**				**<0·001**
Normal	30 (100)	55 (77·5)	6 (24)	
Hepatosplenic	0 (0)	2 (2·8)	3 (12)	
Hepatointestinal	0 (0)	4 (5·6)	4 (16)	
Intestinal	0 (0)	10 (14·1)	12 (48)	
**Right lobe hepatometry**	. (.; .)	6 (6; 14)	6 (4; 6)	0·18
**Left lobe hepatometry**	. (.; .)	6 (5; 8)	6 (4; 6)	0·454
**Hepatometry – ultrasound**	. (.; .)	6 (6; 6)	8 (8; 8)	0·317
**Abdomen**				**<0·001**
Normal	30 (100)	55 (77·5)	6 (24)	
Hollow	0 (0)	1 (1·4)	0 (0)	
Globular	0 (0)	4 (5·6)	1 (4)	
Flat	0 (0)	11 (15·5)	18 (72)	
**Liver**				**<0·001**
Normal	30 (100)	55 (77·5)	6 (24)	
Non-palpable	0 (0)	10 (14·1)	12 (48)	
Palpable	0 (0)	6 (8·5)	7 (28)	

Categorical data expressed as absolute counts and percentages in parentheses. Quantitative data expressed as mean ± standard deviation or as median and interquartile range in parentheses. The chi-square test was used for categorical data, and the ANOVA and Kruskal–Wallis tests were used for quantitative data.

The eggs evaluated by Kato–Katz were elevated in moderate/high group with median of 39 eggs (IQR: 20 – 50) and parasite load between 102 and 1·140 EPG. Clinical findings highlighted that hepatic and abdominal changes were significantly associated with this parasitic load. None of the negative load group had palpable livers, whereas 48% of those with a moderate/high load did, along with altered abdominal ultrasonography findings (*P* < 0·001). Systemic blood pressure and oxygen saturation did not show statistically significant differences across the groups (Table 1).

Laboratory tests focused on levels of ANGs and various blood chemistry markers. BD and TGO levels did not vary significantly between groups, with BD levels averaging 0·26 ± 0·1, 0·23 ± 0·1, and 0·24 ± 0·13, and TGO levels at 27·6 ± 9·9, 25·7 ± 9·1, and 28·1 ± 8 across the negative, low, and moderate/high parasitic load groups, respectively (Table 2).

**Table 2.** Laboratory data and angiopoietin levels related to the parasite load of patients with schistosomiasis

	Parasitic load			
	Negative (*n* = 30)	Low (*n* = 71)	Moderate/high (*n* = 25)	*p-value*
**Direct bilirubin (mg/dL)**	0·26 ± 0·1	0·23 ± 0·1	0·24 ± 0·13	0·301
**AST (TGO) (U/L)**	27·6 ± 9·9	25·7 ± 9·1	28·1 ± 8	0·376
**AST > 38 (U/L)**				0·881
No	27 (90)	66 (93)	23 (92)	
Yes	3 (10)	5 (7)	2 (8)	
**ALT (TGP) (U/L)**	20·1 ± 11·6	20 ± 25·2	18·1 ± 7·5	0·568
**ALT > 40 (U/L)**				0·065
No	27 (90)	70 (98·6)	25 (100)	
Yes	3 (10)	1 (1·4)	0 (0)	
**Total cholesterol (CT) (mg/dL)**	158 ± 35·6	150·4 ± 34·4	149·6 ± 42·2	0·562
**Total cholesterol > 190 (mg/dL)**				0·537
No	24 (80)	62 (87·3)	20 (80)	
Yes	6 (20)	9 (12·7)	5 (20)	
**HDL (mg/dL)**	55·2 ± 9·4	54 ± 10·3	58 ± 11·8	0·428
**HDL < 40 (mg/dL)**				0·463
No	29 (96·7)	67 (94·4)	25 (100)	
Yes	1 (3·3)	4 (5·6)	0 (0)	
**Triglycerides (TG) (mg/dL)**	166·6 ± 140·5	150·3 ± 106·7	113·8 ± 67·3	0·18
**Triglycerides > 150 (mg/dL)**				0·437
No	19 (63·3)	44 (62)	19 (76)	
Yes	11 (36·7)	27 (38)	6 (24)	
**ANG-1 (ng/mL)**	59·3 (48·1; 63·3)	52·8 (44·2; 63·6)	50·9 (42·1; 59·2)	0·174
**ANG-2 (ng/mL)**	0·6 (0·37; 0·96)	0·6 (0·31; 0·93)	1·15 (1; 1·49)	**<0·001**
**ANG-2/ANG-1 ratio**	0·012 (0·005; 0·016)	0·011 (0·006; 0·018)	0·024 (0·016; 0·029)	**<0·001**

Categorical data were expressed as absolute counts and percentages in parentheses. Quantitative data were expressed as mean ± standard deviation or as median and interquartile range in parentheses. The chi-square test was used for categorical data, and ANOVA with Tukey’s post-test and Kruskal–Wallis with Dunn’s post-test were used for quantitative data.

**Figure 1.**
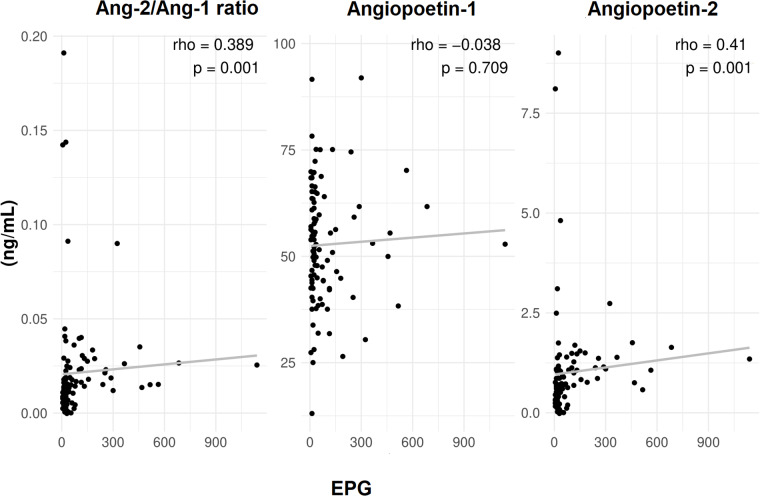
Correlation between eggs per gram of faeces (EPG) with angiopoietins levels in schistosomiasis patients. Spearman's rank correlation coefficient (Rho).

**Figure 2.**
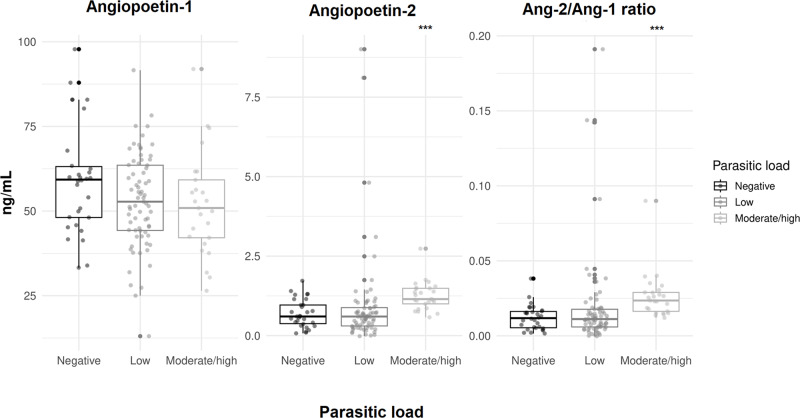
Angiopoietin levels in the serum of schistosomiasis patients, according parasitic load. Box plot representing median and interquartile range, and also a vertical dispersion, with individual value for each patient. Statistical analysis performed by Kruskal–Wallis test with Dunn's post-test. *P* < 0.05*** between moderate to high vs others groups.

Interestingly, a significant positive correlation was observed between EPG with ANG-2 (rho = 0·41, *P* < 0·001) and ANG-2/ANG-1 ratio (rho = 0·389, *P* < 0·001) (Figure 1). Moreover, ANG-2 levels and the ANG-2/ANG-1 ratio were significantly different across the groups (*P* < 0·001), indicating a potential correlation with parasitic load severity. ANG-2 levels were highest in the moderate/high load group at median = 1·15 (IQR: 0·3; 1·49), compared to 0·6 (0·3; 0·89) in the low load group and 0·6 (0·37; 0·96) in the negative group. Similarly, the ANG-2/ANG-1 ratio also increased with higher parasitic loads, being 0·024 (0·016; 0·029) in the moderate/high load group, compared to 0·011 (0·006; 0·018) and 0·012 (0·005; 0·016) in the low and negative load groups, respectively (Table 2, Figure 2).

### Evaluation of ANG levels relation with hepatic and intestinal functions in schistosomiasis patients

Demographic, clinical, and laboratory parameters related to hepatic and intestinal functions were explored in relation to ANG-1 and ANG-2 levels, as well as the ANG-2/ANG-1 ratio in schistosomiasis patients. Analysing key biochemical indicators such as total TGO, total TGP, cholesterol levels (CT and HDL), TGs, palpability of the liver, and with disease classification ranging from normal to hepato-intestinal conditions.

ANG-1 levels remained relatively consistent across various biochemical thresholds and liver palpability, indicating no significant relationship with these clinical parameters. The median ANG-1 levels were similar across groups with abnormal TGO, TGP, cholesterol, and varying TG levels. Moreover, ANG-1 levels did not significantly differ among normal, hepato-splenic, and intestinal categories in schistosomiasis (Table 3).

**Table 3.** Changes related to liver and intestinal function, and clinical changes and the relationship with angiopoietin-1 and 2 levels

	ANG-1		ANG-2		ANG-2/ANG-1 ratio	
	Median (IQR)	*p-value*	Median (IQR)	*p-value*	Median (IQR)	*p-value*
**AST > 38 (U/L)**		0·996		0·065		0·154
No	53·5 (44·3; 62·6)		0·72 (0·41; 1·15)		0·015 (0·008; 0·023)	
Yes	55·3 (45·8; 63·5)		0·49 (0·27; 0·75)		0·011 (0·006; 0·014)	
**ALT > 40 (U/L)**		0·323		0·297		0·636
No	53·5 (44·2; 62·7)		0·71 (0·41; 1·13)		0·014 (0·007; 0·022)	
Yes	59·1 (52; 73·9)		1·03 (0·71; 1·3)		0·015 (0·011; 0·022)	
**Total cholesterol > 190 (mg/dL)**		0·818		0·431		0·709
No	54 (44·2; 63·3)		0·72 (0·41; 1·15)		0·014 (0·008; 0·022)	
Yes	51·4 (46·3; 59·9)		0·57 (0·33; 1·05)		0·013 (0·006; 0·019)	
**HDL < 40 (mg/dL)**		0·827		0·851		0·741
No	53·9 (44·3; 63·3)		0·71 (0·41; 1·13)		0·014 (0·008; 0·022)	
Yes	59·7 (51·6; 59·9)		0·89 (0·25; 1·15)		0·015 (0·004; 0·019)	
**Triglycerides > 150 (mg/dL)**		0·248		0·028		**0·02**
No	52·9 (43·8; 61·3)		0·74 (0·55; 1·15)		0·015 (0·009; 0·024)	
Yes	56·6 (46·3; 63·8)		0·52 (0·29; 1·07)		0·011 (0·005; 0·018)	
**Liver**		0·246		0·158		0·055
Normal	55 (45·2; 63·6)		0·63 (0·36; 1·07)		0·013 (0·006; 0·019)	
Non-palpable	49·4 (42·1; 58·7)		1·03 (0·6; 1·28)		0·017 (0·012; 0·024)	
Palpable	55·5 (44·8; 63·5)		0·76 (0·51; 1·35)		0·015 (0·009; 0·025)	
**Disease classification**		0·179		0·122		0·027*
	55 (45·2; 63·6)		0·63 (0·36; 1·07)		0·013 (0·006; 0·019)	
Hepatosplenic	50·6 (44·8; 55·5)		1·45 (0·76; 1·5)		0·029 (0·025; 0·031)	
Hepatointestinal	62·6 (45·6; 74·8)		0·65 (0·37; 1·14)		0·012 (0·008; 0·017)	
Intestinal	49·4 (42·1; 58·7)		1·03 (0·6; 1·28)		0·017 (0·012; 0·024)	

Quantitative data expressed as median and interquartile range in parentheses. The Mann-Whitney test was used for comparisons between two groups and the Kruskal-Wallis test with Dunn's post-test for comparisons of more than two groups.

* *p* < 0.05 between hepatosplenic vs. other groups.

**Figure 3.**
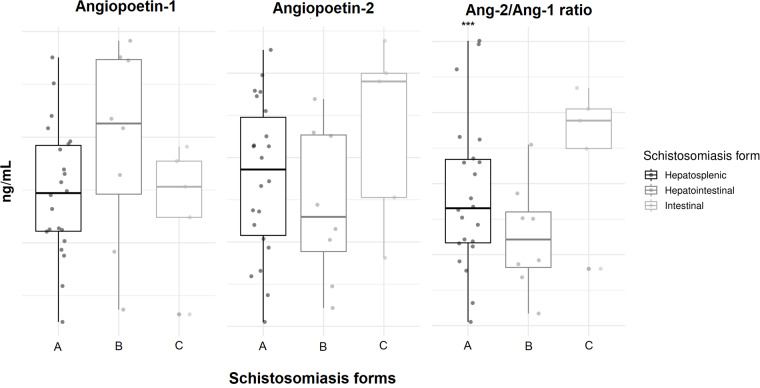
Angiopoietin levels in the different clinical forms of schistosomiasis. Box plot representing median and interquartile range, and also a vertical dispersion, with individual value for each patient. (A) Schistosomiasis hepatoesplenic, (B) schistosomiasis hepatointestinal, (C) schistosomiasis intestinal. Statistical analysis performed by Kruskal–Wallis test. *P* < 0·05***.

Regarding ANG-2 levels, there was observed a significant difference only in patients with elevated TGs (TG > 150), where low median levels were present in this group (*P* = 0·028), suggesting a potential link between lipid metabolism and ANG-2 expression. However, other parameters such as elevated liver enzymes (TGO and TGP), cholesterol levels, and liver palpability did not significantly associated with ANG-2 levels. Finally, The ANG-2/ANG-1 ratio was elevated in the hepato-splenic classification of schistosomiasis disease (*P* = 0·027) (Table 3, Figure 3).

## Discussion

In this study, we aimed to explore the relationships between parasitic load in schistosomiasis and levels of ANGs, as well as various biochemical and clinical parameters. ANG evaluation, such as ANG-2 and its ratio with ANG-1 were associated with elevated parasitic load, particularly in relation to lipid levels and severe hepatic conditions, potentially serving as indicators of underlying pathophysiological changes in schistosomiasis. These findings suggest that ANG-2 levels and the ANG-2/ANG-1 ratio may serve as potential biomarkers for assessing the severity of schistosomiasis.

In the present study, there was a significant difference in racial composition across the parasitic load groups, with an increased prevalence of mixed-race and Black individuals in the moderate/high load group. This finding suggests that certain racial demographics may be more susceptible to higher parasitic loads, possibly due to socio-economic or environmental factors influencing exposure. Studies have shown that low socioeconomic status, which often correlates with poor living conditions and limited access to clean water and sanitation, significantly increases the risk of parasitic infections like schistosomiasis (Rinaldo et al., 2021). Furthermore, the impact of rapid urbanization and the resultant informal settlements with inadequate sanitation and waste management exacerbate the transmission risks of schistosomiasis in urban and peri-urban áreas (Klohe et al., 2021).

Clinically, the moderate/high parasitic load group exhibited a significantly higher prevalence of palpable livers and abnormal abdominal ultrasound findings compared to those with low or negative parasitic loads. Increased parasitic load correlates with progressive hepatic damage and fibrosis, resulting in detectable changes such as hepatomegaly (enlarged liver) and characteristic ultrasound findings like periportal fibrosis and splenomegaly (Hu et al., 2021). These changes are caused by the deposition of parasite eggs in the liver, leading to chronic inflammation, granuloma formation, and fibrosis. This aligns with the well-established notion that increased parasitic load correlates with progressive hepatic damage and fibrosis. However, in this present study systemic blood pressure and oxygen saturation remained unaffected across the groups, possibly indicating that schistosomiasis-related hepatic changes do not severely impair these physiological parameters. This suggests that the primary physiological impacts of schistosomiasis are confined to the hepatic and portal systems rather than causing widespread systemic physiological disruptions (Kamdem et al., 2018).

For the first time, in this present study a significant association of ANG-2 levels and the ANG-2/ANG-1 ratio with increasing parasitic load in schistosomiasis patients was observed. ANG-2 plays a significant role in promoting inflammation and increasing vascular permeability, which are key factors in the progression of schistosomiasis-related vascular changes (Saharinen et al., 2008). Elevated ANG-2 levels are associated with endothelial cell activation, leading to increased vascular permeability and destabilization, particularly under inflammatory conditions (Korhonen et al., 2016). In the context of schistosomiasis, higher levels of ANG-2 disrupt the protective effects of ANG-1 on the vascular endothelium, exacerbating the inflammatory and fibrotic responses seen with increased parasitic burden (Gillen et al., 2019). This imbalance can lead to the characteristic vascular changes observed in schistosomiasis, including enhanced permeability and fibrosis.

Moreover, the ANG-2/ANG-1 ratio, being significantly elevated in the moderate/high group, further supports the hypothesis of endothelial dysfunction and angiogenic imbalance driven by parasitic infection. The ANG-2/ANG-1 ratio is a critical indicator of endothelial dysfunction and angiogenic imbalance (Korhonen et al., 2016; Mota et al., 2021). Thus, the findings of elevated ANG-2 and the ANG-2/ANG-1 ratio in the moderate/high parasitic load group further substantiate the role of these biomarkers in the pathophysiology of schistosomiasis-related vascular damage.

Interestingly, ANG-2/ANG-1 ratio was significantly associated with severe form of schistosomiasis, the hepato-splenic form. Angiogenesis plays a dual role in schistosomiasis, contributing to both fibrogenesis and fibrosis degradation. It is involved in periovular granuloma formation and periportal fibrosis genesis, but also aids fibrosis regression after curative treatment. During fibrogenesis, angiogenesis attracts pericytes, which transform into myofibroblasts, key cells in extracellular matrix formation. Post-treatment, actin-containing pericytes appear in tissue remodelling, especially in repairing vascular lesions (Andrade and Santana, 2010). Hence, we hypothesized that in more severe hepatic conditions, the balance between ANG-2 and ANG-1 shifts, possibly reflecting a more pronounced inflammatory or angiogenic response (Kim et al., 2005). Additionally, the elevated ANG-2/ANG-1 ratio in hepato-splenic schistosomiasis further illustrates the role of ANGs in the progression of hepatic fibrosis and vascular remodelling.

This study had limitations, mostly due to small sample size, which constrained the statistical power and prevented the performance of multivariate analyses. This limitation affects the generalizability of our findings and the ability to control for potential confounding factors. Additionally, the study’s cross-sectional design restricts the ability to infer causal relationships between parasitic load, ANG levels, and clinical outcomes.

In conclusion, ANG levels and their ratios in the blood of schistosomiasis patients were related with elevated parasitic load and severe form of the disease and had potential use as biomarkers for detection of severe hepatic disorder. Moreover, the evaluating of ANGs in schistosomiasis patients contribute to understanding the vascular pathology of schistosomiasis. Further investigations could elucidate the precise mechanisms through which parasitic load influences ANG expression and how these markers can be leveraged for early diagnosis and therapeutic interventions.
