# Angiopoietins as biomarkers of schistosomiasis severity: a cross-sectional study – CORRIGENDUM

**DOI:** 10.1017/S0031182025101224

**Published:** 2025-10

**Authors:** Rachael Lima Sobreira Coimbra, Gdayllon Cavalcante Meneses, Rosângela Lima de Freitas Galvão, Marta Cristhiany Cunha Pinheiro, Luciana Maria de Oliveira, Nicole Coelho Lopes, Bruna Viana Barroso Martins, Letícia Machado de Araújo, Rebeca Yasmin Ribeiro Vieira, Reinaldo Barreto Oriá, Alice Maria Costa Martins, Elizabeth de Francesco Daher, Ângela Maria da Silva, Fernando Schemelzer de Moraes Bezerra

The authors apologise for the inclusion of an error made in the spelling of a co-author’s name in the above article. The author’s name was published as ‘Nicole Coelho Lope,’ but this should be ‘Nicole Coelho Lopes.’

The authors apologise for this error.
